# Thermometers

**Published:** 1875-05

**Authors:** 


Thermometers. — Will not some manufacturer come out with a new thermometer? The affairs in common use in the United States, based upon the plan adopted so long ago by Fahrenheit, are foolish and arbitrary. The wonder is, how sensible people have ever consented to get along with them for a single year.

One trouble is that the zero point is fixed without the slightest reference to thermal conditions. Old Fahrenheit thought 32 deg. below the freezing point of water would be a good place to put a zero mark, so he put it there. The result is that zero means nothing; refers to no special condition of temperature, because it is entirely arbitrary. To say that the mercury is at zero expresses only the idea that the temperature is a good deal colder than it need be to freeze fresh water—32 deg. colder, in fact. Exactly how much colder this would make it, nobody can say.

The same comment may be made with reference to the boiling point of water. Fahrenheit found that with freezing at 32 deg. below, boiling occurred at 212 deg. above, and he was content. But this figure was wholly accidental, depending, as it did, upon a starting point of 32 deg. below for freezing. If he had called 30 deg. below, his freezing point, his boiling point would have been found at 210 deg. above, which would have had the advantage at least of being on the decimal plan.

But what we want, and what some thermometer-maker ought to get rich by supplying, is a measurer of temperature which shall call the freezing point of fresh water at sea-level, zero, and which shall also place boiling fresh water at sea level at, say 100 deg., dividing the range between into equal parts. This would be proceeding with something like method, and the new instruments ought to sell everywhere, because their record would mean something which everybody could comprehend.

---

We have tried the little sweet pellets which constitute the "Van D[illegible]ren remedy" in some cases of indigestion in infants, and have been charmed with the effect. The discharges become natural in a day, and the little sufferers brighten up speedily. A phial of the pellets ought to be in every house where there are children.

The healthy looks of great beer-drinkers are gross deceptions.

